# Ultrasonographic shear wave elastography of the thyroid in patients with sickle cell anemia

**DOI:** 10.2478/abm-2022-0017

**Published:** 2022-06-30

**Authors:** Gulen Burakgazi, Gul Ilhan, Oguzhan Ozcan, Emre Dirican

**Affiliations:** Department of Radiology, Faculty of Medicine, Mustafa Kemal University, Antakya, Hatay 31100, Turkey; Department of Hematology, Faculty of Medicine, Mustafa Kemal University, Antakya, Hatay 31100, Turkey; Department of Biochemistry, Faculty of Medicine, Mustafa Kemal University, Antakya, Hatay 31100, Turkey; Department of Biostatistics, Faculty of Medicine, Mustafa Kemal University, Antakya, Hatay 31100, Turkey

**Keywords:** anemia, sickle cell, anti-thyroid autoantibodies, elasticity imaging techniques, thyroid gland

## Abstract

**Background:**

Although thyroid radiology has been conducted in patients with sickle cell anemia (SCA), to our knowledge, there is no report of thyroid gland assessment using ultrasonographic shear wave elastography (US-SWE).

**Objectives:**

To determine values for ultrasonographic US-SWE of the thyroid in patients with SCA and correlations between thyroid elasticity and biochemical variables used to evaluate thyroid function.

**Methods:**

Prospective case–control observational study of 36 patients with SCA and 33 healthy volunteer controls. US-SWE measurements of thyroid gland parenchyma and biochemical parameters of the participants were obtained and compared, and the diagnostic accuracy of elasticity was determined.

**Results:**

The thyroid volume was smaller in patients with SCA than that in controls (*P* = 0.001). Compared with the controls, the patients with SCA had significantly lower serum levels of free triiodothyronine (fT3) (*P* = 0.004) and thyroglobulin (Tg) (*P* = 0.001) and significantly higher levels of thyroid-stimulating hormone (*P* = 0.028). Thyroid stiffness was significantly higher in the left lobe (LL) of the patients with SCA than in the controls (*P* = 0.003). In the patients with SCA, we found a significant correlation between right lobe (RL) and LL stiffness and serum levels of Tg (RL [*r* = −0.439] and LL [*r* = −0.484]; *P* = 0.021) and fT3 (RL [*r* = −0.463] and LL [*r* = −0.386]; *P* = 0.012). Receiver operating characteristic (ROC) curve analysis of thyroid elasticity that represented a diagnosis of SCA found a cutoff of >7.31 kPa, a sensitivity of 52.0%, and a specificity of 72.0% for the RL (*P* = 0.316, area under the curve [AUC] 0.570), and a cutoff of >8.06 kPa, a sensitivity of 58.0%, and a specificity of 84.0% for the LL (*P* = 0.011, AUC 0.680).

**Conclusions:**

US-SWE can be used to follow up thyroid changes in patients with SCA.

Sickle cell anemia (SCA) is a common multisystemic disease among the hereditary hemoglobinopathies and is characterized by abnormal erythrocytes with a glutamic acid-to-valine missense mutation in the β chain of hemoglobin [1]. Delayed puberty, short stature, and low bodyweight related to thyroidal and gonadal hypofunction can be observed in individuals with SCA in their second and third decades of life. Individuals with SCA have higher serum levels of thyroid-stimulating hormone (TSH) and lower levels of free thyroxin (fT4) and triiodothyronine (fT3) because of primary thyroid insufficiency [2, 3].

Ultrasonography is a noninvasive imaging method commonly used to measure the size of the thyroid gland and to assess and monitor diffuse thyroiditis and nodular parenchymal and fibrotic conditions of the thyroid gland [4]. Shear wave elastography (SWE) is an imaging method used to evaluate tissue elasticity quantitatively in regions of interest. SWE uses an acoustic radiation force to generate low-frequency (about 50 Hz) shear waves that displace tissue. Tissues can be differentiated as soft (elastic) or stiff (decreased elasticity) by measuring the speed of shear wave propagation using Doppler ultrasonography. During the measurement, soft tissues manifest as blue, while stiffer tissues manifest as red, in color-coded images [5]. Quantitative information about the elasticity of tissues can be obtained using the SWE method during ultrasonographic shear wave elastography (US-SWE) examinations [6]. SWE values correlate with the elasticity of a given tissue element. The harder the tissue, the higher the SWE values and lower its elasticity; and the softer the tissue, the lower the SWE values and higher its elasticity [7]. Because SWE can demonstrate tissue elasticity, it has been used to show parenchymal changes including fibrosis in a variety of tissues, including liver [8]. Basal SWE values have been determined for a wide variety of tissues and parenchymal lesions using US-SWE [8, 9, 10]. The stiffness of the thyroid gland is determined by the structural features of its tissue matrix, such as its cells, membranes, extravascular matrix, and microvasculature. While it is the microscopic structure that determines the echo in ultrasonography, the values obtained by US-SWE pertain to the histological tissue structure. Thus, important information differentiating normal tissue from thyroid gland nodules and diffuse parenchymal diseases can be obtained using US-SWE imaging [11].

Patients with SCA develop primary thyroid insufficiency [1, 2], which presents on ultrasonography as a decreased volume of the thyroid gland and increased resistive index in the thyroid parenchyma on Doppler ultrasonography [12]. Although radiological studies of the thyroid have been conducted in children and adults with SCA, to our knowledge, there is no study in which US-SWE was used to assess the thyroid gland in patients with SCA.

Here, we aimed to determine the parenchymal elasticity of the thyroid gland in patients with SCA using US-SWE, its diagnostic accuracy, and correlations between the US-SWE findings and biochemical parameters used to evaluate thyroid function.

## Methods

### Patients and imaging procedures

Patients with SCA who were admitted to our institute from January 2016 to January 2017 and age- and sex-matched healthy controls were included in this prospective case–control study. STARD 2015 reporting guidelines were followed [13]. The study was approved by the ethics committee, Faculty of Medicine, Mustafa Kemal University, and was conducted according to the principles of the Declaration of Helsinki and its contemporary amendments (2013). Documented informed consent was obtained from all participants.

Individuals who had a history of thyroid surgery, thyroid nodules, or heterogeneous thyroid parenchyma were excluded from the study. Patients with SCA who were in pain crises or had a history of blood transfusion within the previous month were also excluded from the study due to possible variations in their biochemical values and parenchymal measurements secondary to the increase in acute-phase reactants and sudden increase in ferritin levels.

The same radiologist evaluated the thyroid gland of all patients and healthy control participants who were referred to the radiology department using the linear probe (4–10 MHz) of a Logiq E9 XDclear ultrasound imaging system (GE Health-care). The participants were examined while they were supine by applying gel to their necks at room temperature.

**Figure 1:**
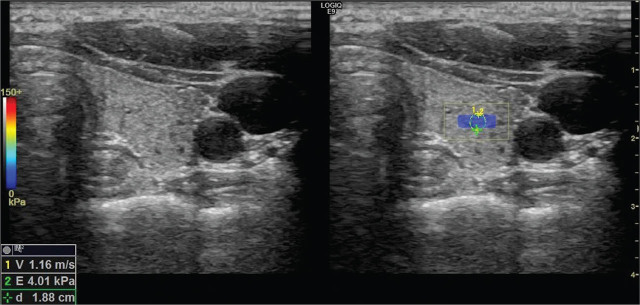
SWV measurement in a healthy patient in the control group generated by the acoustic radiation force impulse. SWV (1.16 m/s) reflects tissue elasticity, which can be calculated using Young's modulus. Thus, shear wave elasticity can be used to evaluate tissue stiffness, both quantitatively and objectively. Parenchymal stiffness measured in the ROI on the left lobe was 4.01 kPa. ROI, region of interest; SWV, shear wave velocity.

**Figure 2:**
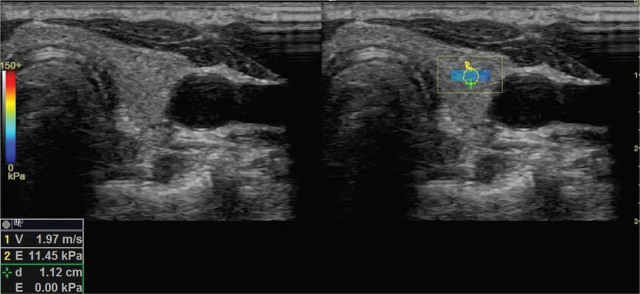
Shear wave velocity measurement in a patient with SCA. Parenchymal stiffness measured in the ROI on the left lobe was 11.45 kPa. ROI, region of interest; SCA, sickle cell anemia.

After the measuring the volume of thyroid gland parenchyma, isthmus, and the right and left lobe, SWE measurements were obtained using a linear probe without applying any compression while the patient was holding their breath. The radiologist obtained 3 different measurements from the right and left lobe in the axial plane, being distant from the vascular structures with a standard region of interest (ROI) of 1.2–1.5 mm while the probe was perpendicular to the parenchyma. Velocity and elasticity values were measured from the right and left lobe of the thyroid gland separately at each time with a constant ROI, and the mean SWE value was calculated from the 3 measurements (**Figures 1 and 2**).

Morning fasting blood samples of the patients and healthy control participants were collected by vein puncture. All blood samples were centrifuged at 1500 ×*g* for 10 min, at 4°C. Serum levels of TSH, fT4, free triiodothyronine (fT3), thyroid peroxidase antibody (TPOAb), and thyroglobulin antibody (TgAb) were determined with an electrochemiluminescence method using an ADVIA Centaur XP immunoassay system for hormone analysis (Siemens Diagnostics).

### Data collection and statistical analysis

Power analysis for the study was conducted with G Power software (release 3.1.9.2). A medium effect size (Cohen's d 0.50), 0.95 power, and 0.05 margin of error (*P* < 0.05) were used to determine the number of people from retrospective scanning of the sample group in the study. Continuous variables are expressed as mean ± SD, and categorical variables are expressed as n (%). The distribution of data was checked using a Shapiro–Wilk test. Outlier values in the data set, detected using a boxplot, were excluded from the analysis. A Student *t* test, Mann–Whitney *U* test, and Pearson and Spearman rank correlation analyses were used to evaluate the relationships between thyroid elasticity and biochemical variables. Receiver operating characteristic (ROC) curve analysis was used to determine the cutoff of the SWE measurements that represent a diagnosis of SCA. The area under the curve (AUC), sensitivity, and specificity were calculated. *P* < 0.05 were considered significant. IBM SPSS Statistics for Windows (version 21.0) was used for all analyses.

## Results

Demographic and laboratory findings of the study population are shown in **Table 1**. We included 36 patients with SCA (18 male, 18 female) and 33 healthy volunteer control participants (17 male, 16 female) in the present study. The groups were homogenous in terms of sex and age. There was no significant difference between the mean age of patients in the SCA group (31.3 ± 8.2 years) and healthy control group (32.6 ± 5.8 years) (*P* > 0.05). Body mass index (BMI) values were within normal limits in both groups; despite that there was a significant difference in BMI values between the groups (*P* = 0.004). The thyroid function was within normal limits in all patients. Most (28) of our patients had received hydroxyurea treatment, and 5 received iron chelator treatment. However, 25 of our patients received erythrocyte suspension, and the average number of transfusions was 3.16. Thyroid volume in the patients with SCA was significantly less than that in the controls (*P* = 0.001). Compared with the healthy controls, there was a lower level of fT4 in patients with SCA, but the difference between the groups was not significant. Compared with the control participants, serum levels of fT3 and thyroglobulin (Tg) were significantly lower (*P* = 0.004 and *P* = 0.001, respectively) and serum levels of TSH were significantly higher (*P* = 0.028) in the patients with SCA. We found no significant difference in levels of TgAb and TPOAb between the groups (**Table 1**).

**Table 1:** Demographic, blood, and serum biochemical variables

		**SCA (n = 36)**		**Healthy controls (n = 33)**		***P***
	
		**Mean ± SD**	**Median**	**Mean ± SD**	**Median**	
Age (years)		31.3 ± 8.2	30.5	32.6 ± 5.8	32.0	0.46
Sex, n (%)	Female	18 (52.9)		16 (47.1)		0.90
	Male	18 (51.4)		17 (48.6)		
BMI (kg/m^2^)		21.30 ± 1.83	22.00	22.78 ± 2.25	23.00	0.004^*^
Volume (mL)		3.91 ± 1.39	3.45	5.25 ± 1.84	4.80	0.001^*^
Left lobe		3.25 ± 1.21	2.90	4.26 ± 1.27	4.20	0.001^*^
Isthmus (mm)		3.06 ± 1.24	2.95	3.29 ± 0.79	3.00	0.35
Thyroid stiffness (kPa)						
Right lobe		7.39 ± 3.30	7.57	6.39 ± 1.82	6.34	0.13
Left lobe		8.20 ± 2.89	8.43	6.41 ± 1.80	6.45	0.003^*^
fT3 (pmol/L)		2.92 ± 0.61	2.91	3.27 ± 0.44	3.16	0.004^*^
fT4 (ng/dL)		1.29 ± 0.16	1.29	1.30 ± 0.16	1.32	0.75
TSH (mU/L)		1.82 ± 0.80	1.68	1.44 ± 0.55	1.30	0.028^*^
Tg (ng/mL)		5.81 ± 3.04	5.59	13.55 ± 7.76	10.55	0.001^*^
TgAb (IU/mL)		21.36 ± 6.04	20.00	21.95 ± 10.42	20.00	0.47
TPOAb (IU/mL)		23.41 ± 16.15	20.00	20.00 ± 0.00	20.00	0.82
Ferritin (ng/mL)		656.3 ± 622.6	426.6			
Hb (g/dL)		8.88 ± 1.75	8.75			
Hct (%)		26.11 ± 5.12	26.35			
CRP (mg/L)		10.08 ± 7.94	7.18			

* *P* < 0.05.

BMI, body mass index; CRP, C-reactive protein; fT3, free triiodothyronine; fT4, free thyroxin; Hb, hemoglobin; Hct, hematocrit; SCA, sickle cell anemia; Tg, thyroglobulin; TgAb, thyroglobulin antibody; TPOAb, thyroid peroxidase antibody; TSH, thyroid-stimulating hormone.

The thyroid gland US-SWE stiffness was higher (7.39 ± 3.30 kPa right lobe (RL and 8.20 ± 2.89 kPa left lobe (LL) in the patients with SCA than in the healthy control participants (6.39 ± 1.82 kPa RL and 6.41 ± 1.80 kPa LL), but only the LL stiffness was significantly higher in the patients with SCA (*P* = 0.003). We found no correlation between the TPOAb levels and thyroid elasticity (**Table 1**).

**Figure 3:**
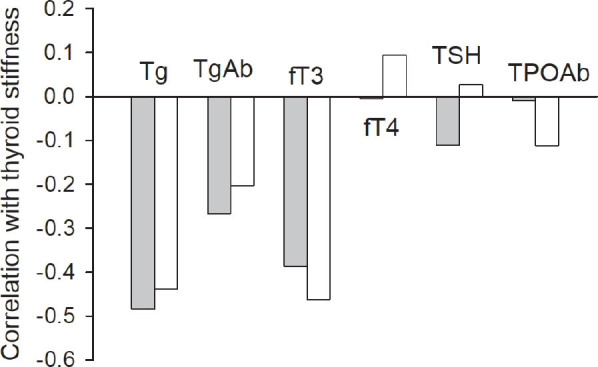
Correlations of serum levels of thyroid function molecules and antibodies with thyroid stiffness left (gray bars) and right (white bars). Significant correlations were found between serum levels of Tg fT3 and left (*P* = 0.008 and *P* = 0.024, respectively) and right stiffness (*P* = 0.020 and *P* = 0.007, respectively). Other variables, TgAb, fT4, TSH, and TPOAb, were not significantly correlated. fT3, free triiodothyronine; fT4, free thyroxin; Tg, thyroglobulin; TgAb, thyroglobulin antibody; TPOAb, thyroid peroxidase antibody; TSH, thyroid-stimulating hormone.

The negative correlation between levels of TgAb and thyroid stiffness in patients with SCA was not significant (*P* > 0.05, *r* = −0.203 RL and *r* = −0.226 LL). Nor did we find any significant correlation between the thyroid volume and the LL stiffness in the patients with SCA (*r* = −0.222, *P* = 0.20). However, in the patients with SCA, we did find a significant correlation between RL and LL stiffness and serum levels of Tg (*r* = −0.439 RL and *r* = −0.484 LL, *P* = 0.021), and levels of fT3 (*r* = −0.463 RL and *r* = −0.386 LL, *P* = 0.012) (**Figure 3**).

**Figure 4:**
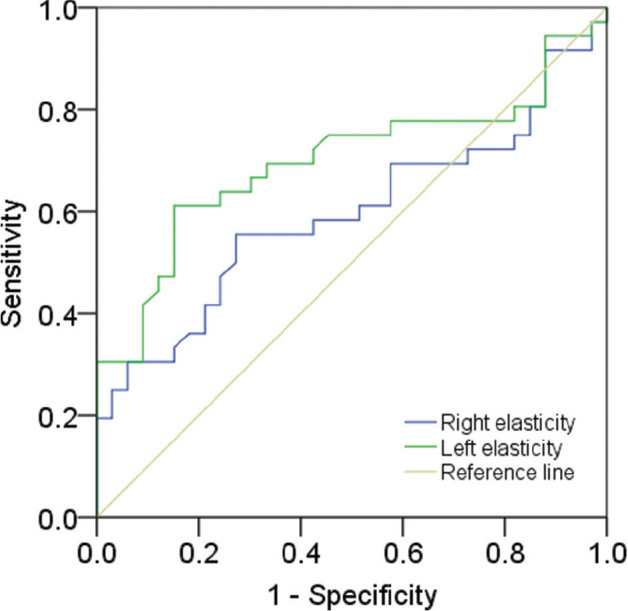
ROC curve of thyroid gland SWE in patients with SCA. The cutoff for the RL elasticity (blue line) is 7.31 kPa (AUC 0.57, *P* = 0.32), and the cutoff value for the LL elasticity (green line) is 8.06 kPa (AUC 0.68, *P* = 0.011). Reference line is tan. AUC, area under the curve; LL, left lobe; RL, right lobe; ROC, receiver operating characteristic; SCA, sickle cell anemia; SWE, shear wave elastography.

The ROC curve of thyroid elasticity in the patients with SCA showed a cutoff with the highest diagnostic accuracy for SCA of >7.31 kPa, a sensitivity of 52.0%, and a specificity of 72.0% for the RL (*P* = 0.316, AUC 0.570), and a cutoff of >8.06 kPa, a sensitivity of 58.0%, and a specificity of 84.0% for the LL (*P* = 0.011, AUC 0.680) (**Figure 4**).

## Discussion

In patients with SCA, US-SWE indicated that thyroid gland stiffness was high and negatively correlated with serum levels of Tg and fT3.

Hypoxia, hemolytic anemia, and changes in endocrine tissues secondary to recurrent transfusion are observed in the patients with SCA. The pathophysiology of these changes and the etiology of thyroid dysfunction are not fully known. In some studies, autopsy results revealed significant iron accumulation in the thyroid, which suggests that the etiology of the primary thyroid failure may be transfusional hemosiderosis and cellular damage in the thyroid because of transfusional hemosiderosis [3, 14]. The low elasticity values found in our study may be a result of the transfusional hemosiderosis and the fibrosis developing at the cellular level.

Except for the Doppler ultrasonography study conducted by Karazincir et al. [12], we did not find any radiological studies in patients with SCA. These investigators also found higher resistive and pulsatility indices of the thyroid and a lower thyroid volume in patients with SCA than in controls, and suggested that this may be secondary to changes in micro-circulation. Similarly, in the present study, we found a lower thyroid volume in the patients with SCA than in the healthy control participants.

Bankir et al. [15] found greater stiffness of the thyroid gland in patients with acromegaly associated with higher levels of insulin growth factor-1. In the present study, we found a negative correlation between stiffness and thyroid volume; however, it was not significant. The lack of correlation may be because US-SWE cannot show histological changes before the microscopic changes have been observed in the thyroid gland parenchyma [8].

Various cutoff values for thyroid disease diagnosis have been reported in studies of the thyroid. Kandemirli et al. [4] reported 12.3 kPa as a cutoff in patients with pediatric Hashimoto thyroiditis. Uysal and Öztürk [16] reported 6.38 ± 1.97 kPa (RL) and 8.81 ± 3.00 kPa (LL) as cutoffs in healthy children. Arda et al. [5] reported 10.97 ± 3.1 kPa as the cutoff in healthy adults. In the present study, we found cutoffs of 7.31 kPa (RL) and 8.06 kPa (LL) associated with a diagnosis of SCA.

Smiley et al. [14] reported that the stiffness in the thyroid may be as a result of fibrosis that develops due to the diffuse iron accumulation in the thyroid parenchyma of patients with SCA. We found lower thyroid elasticity in the patients with SCA than in the healthy control participants. This may be due to iron accumulation in the thyroid parenchyma.

Tg is synthesized only by the thyroid follicular cells and thus the serum level of Tg reflects the thyroid tissue load [17]. We found a negative correlation between serum levels of Tg and thyroid stiffness. This may be indicative of the fibrotic changes in the thyroid gland at the cellular level.

Low fT3 with normal fT4 levels may be due to impaired environmental conversion of fT4 to fT3. It may not reflect thyroid function. High TSH may indicate subclinical thyroid hypofunction. Hagag et al. [18] found higher serum levels of TSH and lower levels of fT3 and fT4 in children with SCA. Similarly, we found a significantly higher serum levels of TSH and significantly lower levels of fT3 and Tg in the patients with SCA than in the healthy control participants. These levels may be an indication of the thyroid insufficiency that develops because of the parenchymal accumulation of iron. Hagag et al. [18] reported that there was no significant difference between TgAb and TPOAb values in pediatric patients with SCA and those without. Similarly, we did not find any significant difference in levels of TgAb or TPOAb between adult patients with SCA and the healthy controls. However, the levels of TPOAb tended to be higher in the patients with SCA than in the controls. This suggests that the elevated antibodies in autoimmune diseases and thyroid insufficiency with SCA may be associated with parenchymal iron accumulation, rather than with autoimmunity.

Kandemirli et al. [4] found a significant positive correlation between thyroid stiffness and serum levels of TPOAb values in children with thyroiditis. However, they did not find any significant relationship between thyroid elasticity and levels of TgAb. Fukuhara et al. [19] found a weak positive correlation between thyroid stiffness and levels of TPOAb in diffuse autoimmune thyroid disease. However, they did not find any significant association between thyroid tissue stiffness and serum levels of TgAb. These findings are considered to reflect the chronic immune response of TPOAb and early immune response of TgAb. We found lower serum levels of Tg and higher levels of TPOAb in the patients with SCA than in the healthy controls, but no significant relationship between thyroid elasticity and the serum levels of TgAb and TPOAb. The sensitivity of US-SWE may not be sufficient to indicate damage as a result of the parenchymal iron accumulation in the patients with SCA.

We found BMI values to be within normal limits in both patients and healthy controls, although there was a significant difference between the groups. A significant relationship between thyroid elasticity and weight, height, and age has been reported [20]. However, we found no significant difference between the thyroid US-SWE values measured in women and men, possibly because our small sample size did not provide sufficient power to detect a difference. Significant differences may be obtained in studies with a larger sample in which sexes are homogeneously distributed.

Our study has some limitations. The sample was small and obtained only from a single center. The thyroids in the patients were not assessed histopathologically. The US-SWE measurements were only made in the axial plane. Nevertheless, our study was conducted prospectively in a specific patient group and provides information about thyroid volume and function by measuring thyroid elasticity in patients with SCA, without the need for biopsy.

## Conclusions

Thyroid stiffness was higher in the patients with SCA than in the healthy controls. We found no significant association between the elasticity of the thyroid parenchyma and serum levels of TPOAb of TgAb in the patients with SCA. The reason for the lower thyroid parenchymal elasticity in patients with SCA may be parenchymal iron accumulation. In patients with SCA who undergo various invasive procedures (predominantly recurrent blood transfusions) during diagnosis, treatment, and follow-up, thyroid follow-up can be performed with a noninvasive US-SWE, thus reducing the overall number of invasive procedures that the patient needs to undergo. Further studies with histopathological evaluations using a larger number of patients are required to demonstrate the utility of this method to evaluate thyroid parenchymal changes in patients with SCA.
